# The Hospital Nursing Supplement

**Published:** 1895-06-01

**Authors:** 


### "The Hospital" Nursing Mirror.

Being the Extra Nursing Supplement of "The Hospital" Newspaper.

[Contributions for this Supplement should be addressed to the Editor, The Hospital, 428, Strand, London, W.C., and should have the word "Nursing" plainly written in left-hand top corner of the envelope.]

### News from the Nursing World.

### CHELSEA HOSPITAL FOR WOMEN.

The Women's Hospital in the Fulham Road is always bright and airy, and it was seen to special advantage on May 27th, when her Royal Highness the Duchess of Teck thoroughly inspected it. Every room was flooded with sunshine, and a ward was opened by, and named after, her Royal Highness. The training of probationers is being ably organised by the recently appointed matron, Miss M. Heather-Bigg, whose good work as Sister at Charing Cross Hospital, and later as Matron of the Bromley Cottage Hospital, is well known. Miss Heather-Bigg is doing her best with the materials that lie to her hand, but we trust that it will not be long ere a proper nurses' home forms part of the equipment of this hospital, and then single bed-rooms will supersede the present inadequate provision for the nursing staff. The hours on duty are arranged so as to give each nurse and probationer liberal time off duty, and it is evident that their comfort and welfare are duly considered by those under whom they work.

### "THE HOSPITAL" CONVALESCENT FUND.

At this season of the year requests made for the assistance of The Hospital Convalescent Fund are naturally more numerous than in the winter months. We hope that subscribers will keep us informed of any cases of which they have personal knowledge and which they know to be those of genuine working nurses for whom the much-needed change of air is quite impossible without help. The H.C.F., first established as "The Nurses' Bed," was founded to enable tired or delicate nurses to enjoy a stay by the sea, free of expense, when their means did not permit of their otherwise taking a short holiday. The fund has already given to many weary nurses facilities for rest without cost or anxiety. There are no expenses of management, the fund being administered by the honorary treasurer and secretaries. Applications for assistance are made by letter only, and must be addressed "Hon. Secretary, "The Hospital" Convalescent Fund, 428, Strand, London." Candidates would save much time by stating in a clear concise form what kind of change they desire, and whether permanent or temporary benefit be likely to result from it. The address of a doctor or matron to whom the nurse's circumstances are known should also be sent. We are repeatedly obliged to impress upon applicants that the fund exists to aid nurses to regain health and strength for resuming work. Chronic cases and persons who no longer follow the nursing profession are not eligible, as the H.C.F., established by nurses for nurses, cannot be diverted from its original purpose. Hard as it is to refuse all sorts and conditions of women, it must be done when they are obviously ineligible cases. With the increased number of candidates which summer always brings we need new subscriptions. A long time has passed since we last invited our readers to co-operate with us in this matter, but now that rest and change are immediate necessities for many workers it appears a reasonable moment for a fresh appeal to be made. All sums, however small, sent to the hon. secretaries, will be promptly acknowledged through the treasurer.

### A STUDENTS' CONCERT.

The concert annually given by the students of the London Hospital Medical School to the nursing staff and their friends is announced to take place on Thursday, June 6th, and it will doubtless be as successful as all the previous entertainments which have taken place in the fine college library of that institution.

### NURSING IN SOUTH LONDON.

The South London District Nursing Association, which has been in existence about eleven years, is in sore need of financial support. It is affiliated with the Queen's Jubilee Institute, and in the course of last year the nurses paid 24,429 visits to 836 patients. At the recent annual meeting the claims of the association were ably advanced by Canon J. Erskine Clarke, General Cumberland, and other speakers, who represented that unless the funds received considerable augmentation the work could not possibly go on.

### NOTTINGHAM.

In the nineteenth annual report of the Nottingham and Nottinghamshire Nursing Association a steady growth of work is chronicled. The number of district nurses has increased to nine, an additional one being specially engaged when the Board of Guardians voted £60 to the institution to secure attendance on such out-door patients in the town as received parish relief. The whole expense of the district branch of the Nottingham Association is met by voluntary subscriptions, and the work is perfectly unsectarian. That acute as well as chronic cases are admirably tended is proved by seven patients making good recoveries from typhoid under one nurse within the space of three months. The record of other cases requiring skilled care is also good. The private nursing branch of the institution is self-supporting, and out of the balance a gratuity has been distributed, but it does not appear that any portion of the profits are devoted to a pension fund for the nurses. Their salaries have recently been raised to £30 per annum, and it would be well for the committee either to further augment these to allow of the nurses making provision for old age, or else to supplement their earnings, as other institutions do, by taking out policies for them in the Royal National Pension Fund.

### TROUBLE AT MACCLESFIELD.

An application was made by Miss Wingfield, matron of Macclesfield Infirmary, for four months' leave of absence on account of ill-health. At a recent meeting of the governors this request was declined by a majority, who voted for the acceptance of Miss Wingfield's alternative offer of immediate resignation of her office. She had proposed to pay for an efficient substitute in the event of leave of absence being granted, but the House Committee have contented themselves with placing the matron's duties in the hands of a person described by Miss Wingfield as an old nurse and former ward maid in the institution. Since Miss Wingfield's appointment last June, great changes have taken place, the nursing department being entirely reorganised; and on hearing of her resignation, four or five of her nurses refused to remain. The long reports in the local press show the existence of lamentable discord amongst medical and lay officials, and in face of the conflicting statements made at the meeting, it is impossible to form a just opinion on the matter. Miss Wingfield's letter in the *Manchester Courier* contains statements which need to be corroborated or refuted. It will be somewhat difficult for any competent and fully-trained lady to be found willing to carry on the supervision of the nursing department at Macclesfield Infirmary unless she is sure of the co-operation of those with whom she is associated.

### UNANIMOUS BUT UNWISE.

The Kettering Board of Guardians are reported to have unanimously accepted the advice of the master regarding the appointment of Miss Edith Rowlatt, the girls' trainer to the office now vacant in the workhouse through the death of Nurse Gray. It is fortunate that the Local Government Board holds sufficiently wide views of the needs of the sick poor to render it unlikely that they will authorise the appointment of an untrained person. Of course, Miss Rowlatt may be a duly certificated nurse, but it hardly seems likely in that case that she would be content with a post from which advancement to a salary of £20 per annum is "promotion." The Guardians and the master are surely aware that this rate of pay will not secure the services of a competent nurse in the present day.

### UNPAID COMPANIONS.

An action was recently brought at the Belfast Recorder's Court by a monthly nurse, to recover the balance of an account due to her for attendance on a lady. She asserted that she was engaged at twenty-one shillings a-week, and that she was dismissed at the end of three with twenty-five shillings only. In defence it was urged that this sum was sufficient, it being usual "for nurses to go to ladies for a week or so for nothing in order that they (the ladies) might get used to the nurses." At the conclusion of the evidence a decree was given for the balance due. In this case, the process known as "getting used to" the nurse might have proved a singularly unremunerative experience for the latter. Her engagement appears to have terminated whilst her stay with the patient was still liable to be regarded as a visit paid "for nothing."

### CONTRASTED QUALIFICATIONS.

It is considered that a full course of training in general nursing is essential to fit a woman for the post of charge nurse (or sister) in a large fever hospital, and even for assistant nurses at least one year's preliminary training is now demanded, the work of both ranks being, nevertheless, carried on entirely under direct medical supervision. In strange contrast to the qualifications demanded of these nurses stand the Bury Town Council's modest requirements in their little isolation hospital. Of course there will there be neither resident medical nor nursing superintendence of the person whom they appoint. She should, then, be a most competent woman, yet they wish her to combine the duties of nurse and caretaker. She is to be wife of a man who will act as porter and gardener. Her wages will consist in a share of twenty-five shillings per week which the committee fix as the joint salary of the couple, discreetly avoiding any proportioning of the respective value of the wage winners. When the hospital is empty the couple will have an allowance for board, and when there are patients they will be boarded instead. Twenty-five applications have been received, according to the local press reports, and it is to be hoped, for the sake of her future patients, that the nurse-caretaker selected will possess thorough knowledge of the principles of isolation and disinfection, as well as adequate training for the conscientious nursing of cases of infectious diseases.

### A GOOD EXAMPLE.

An appeal for increased subscriptions, made with more or less urgency, so often forms part of notices respecting the work of District Nursing Associations, that it is particularly pleasant to have to record the good fortune of the Dundee Sick Poor Nursing Society. A donation of £500 has just been handed over to the president of this society by Mr. John Kidd, of Dundee, with a request that it should be securely invested, and the free income applied annually to the purposes of the institution.

### SHORT ITEMS.

The Westhampnett Guardians are considering a scheme for superseding pauper attendants by trained nurses in their workhouse infirmary.—The work of Nurse Oddy in connection with the Kendal Home Nursing Association is greatly appreciated in the district.—Under the name of "Cottage Nurses," the Essex Association is prepared annually to supply the public with twelve women, who, after six months' instruction, are considered by the association sufficiently "trained" for the service of the sick poor in their own homes.—At the general annual meeting of the Chipping Norton Nursing Association attention was called to the need for increased local financial support.—The Nelson Board of Guardians decided unanimously at a recent meeting to add two probationers to their present Workhouse Infirmary nursing staff.—The clerk to the Honiton Guardians reported at a recent meeting that £12 10s. had been spent in advertisements, and "again there were no applications for the post of nurse at the workhouse"; it seems probable that an alteration in the form of advertisement as regards the salary offered will have to be considered.—The Llanelly Board of Guardians have subscribed £10 to the District Nursing Association.—The Tiverton District Nurse Association reports a year of useful work.—The annual meeting of the Palestine and Lebanon Nurses' Mission was recently held in Exeter Hall Parlour, the Rev. J. E. Campbell-Colquhoun being in the chair.

### Elementary Anatomy and Surgery for Nurses.

By W. McADAM ECCLES, M.B., M.S., F.R.C.S., Lecturer to Nurses, West London Hospital, &c.

### XIX.—HÆMORRHAGE, AND ITS TREATMENT.

HÆMORRHAGE, or the loss of blood from a blood vessel, is the natural consequence of a wound of such. It would, therefore, occur from (1) arteries, when it is called arterial; (2) veins, when it is styled venous; (3) capillaries, when it is termed capillary.

Arterial hæmorrhage is characterised by blood of a bright red colour issuing in jets or jerks corresponding to each contraction of the heart, although there is no actual cessation of the stream between.

Venous hæmorrhage is distinguished from arterial by the blood being generally of a dark purplish colour, and escaping in a steady stream without any force or pulsation.

Capillary hæmorrhage is of the nature of oozing from a considerable surface, and the blood gradually collects and then trickles away.

Arterial hæmorrhage is divided into three classes: (*a*) Primary, or that which occurs immediately after the wound of an artery; (*b*) Reactionary, or that which is apt to come on as the patient is recovering from the depressing effects of an operation; (*c*) Secondary, or that which happens after the period of reaction is over up to the complete healing of the wound. The treatment of bleeding from an artery is of the utmost importance. It must be prompt and effective, otherwise the patient will soon faint from loss of blood, and often, if the loss be very excessive, will die.

### THE TREATMENT OF PRIMARY ARTERIAL HÆMORRHAGE.

In order to fully appreciate the methods by which a surgeon seeks to control this dangerous variety of bleeding, it is advantageous to have some idea as to the plan which nature adopts in arresting it. There are two stages in her process, the temporary and the permanent arrest. The temporary arrest is brought about by the contraction and retraction of the muscular coat of the blood-vessel, and the clotting of blood about the wound of the artery, as well as within the tube itself. At the same time the force of the heart's beat may be diminished by the faintness which is caused by the loss of blood. Even when these means are in evidence the blood may burst forth again and the patient may lose his life. In order, therefore, to secure the permanent arrest of the hæmorrhage, nature goes on to firmly seal up the wounded artery by plastic inflammation, and if not interrupted by septic processes the result is perfect. Surgical art may step in and aid the work of nature in effecting a temporary cessation of the flow, but it is powerless to take any part in the function of permanently arresting the same. The blood from a wounded artery may be for the time prevented from escaping by pressure applied either to the bleeding point itself, or on the main vessel on the heart or proximal side of the wound. It may be taken as a fact that any hæmorrhage, the source of which can be seen, is comparatively easily controlled for a certain space of time by pressure. Never be tempted to cover over the spot from which the blood issues, unless firm pressure is at the same time applied. A concealed hæmorrhage is merely false security, and will in the end lead to disaster. Pressure by the finger or thumb is probably the most efficient which can be used, and should at once be tried upon the seat of bleeding, especially when this occurs unexpectedly.

Pressure with the fingers cannot be maintained for long, and, therefore, mechanical means have been brought into requisition. The pressure forceps is an indispensable instrument in the present day. Various modifications of the well-known forceps of Sir Spencer Wells are in general use. By their application to the actual wound in a vessel, not only is the blood prevented from issuing forth, but the walls of the artery are so tightly compressed as to be temporarily sealed.

A tourniquet is also an instrument of which a nurse should thoroughly understand the principle. By its means hæmorrhage is prevented, not by pressure at the seat of the wound, but by compression of the main artery of the part on the proximal, or heart side of the injury. For instance, if there be a wound of the forearm involving one or both of the arteries the tourniquet would have to be applied to the brachial artery in the arm, that is, therefore, on the main artery but nearer the heart. Various forms of tourniquets are in use, but of the number the simple elastic band or cord is the most efficient and readily adjustable in cases of emergency. It is frequently called Esmarch's tourniquet. But by far the most satisfactory way of occluding a severed artery is by tying a ligature around it. Silk, of the variety known as Chinese twist, is the material most commonly employed for this purpose. It is highly necessary that it be rendered thoroughly aseptic by adequate boiling, and then soaking in an antiseptic solution, such as 1 in 20 carbolic acid lotion.

### THE TREATMENT OF REACTIONARY ARTERIAL HÆMORRHAGE.

As has been stated, this form of bleeding is liable to come on during the period of reaction which ensues after the depressing influence of an operation. When the wound is closed, many small vessels, including arteries, have ceased to bleed, but as soon as the patient gets comfortable and warm in bed, the heart's action becomes more forcible, and then it is that hæmorrhage from these vessels may occur. In other cases recurrent or reactionary hæmorrhage is due to the slipping of a ligature. The treatment of such a complication resolves itself into prophylactic and actual treatment. Take the case of a stump wound after an amputation; firm pressure by bandaging and elevation of the part will usually obviate the hæmorrhage; but supposing it to have come on, a removal of the dressings and their re-application with an increased amount of pressure will ordinarily terminate the flow. It is seldom necessary to open up the wound and ligature a vessel.

### THE TREATMENT OF SECONDARY ARTERIAL HÆMORRHAGE.

This is an extremely dangerous sequela of a wound happily but seldom seen in the present day. It is hæmorrhage which occurs in the period after reaction is over until the complete healing of the wound. It is most frequent about the sixth to the ninth day. Sepsis is the usual predisposing cause, and ulceration through the coats of an artery the actual factor in its production. There is often warning of the likelihood of its appearance by the dressing being blood-stained, say, on the sixth day after the operation. If this has been noticed everything should be in readiness for prompt treatment should a sudden gush of blood take place. A suitable tourniquet is to be prepared, and the nurse should make certain that she knows exactly where and how to compress the main artery of the part on the first appearance of any hæmorrhage. It is, however, her duty to immediately send for the surgeon, and, pending his arrival, to see that she efficiently controls the bleeding.

A considerable loss of blood—whether venous or arterial—is accompanied by certain constitutional symptoms. Faintness, evidenced by pallor of the face, a feeble or absent radial pulse, and frequently a temporary loss of consciousness, often comes on. Breathing is sighing in character; the pupils dilate, and there is much restlessness. The extremities become cold, and usually there is marked perspiration. Patients in such a condition require very careful treatment. On no account should the head be raised for by so doing the brain may be deprived of the small amount of blood which it is receiving and fatal syncope may follow. On the contrary, the head should be kept horizontal, or even lower than the trunk, and the legs should be raised. If the source of hæmorrhage has been found and the bleeding controlled, stimulant in the form of brandy or ether is good, but it must be remembered that faintness is one of nature's methods to bring about a temporary arrest of hæmorrhage, and if the heart's action be increased while the wound in the vessel is still patent disastrous consequences will ensue.

### Workhouse Infirmary Nursing Association.

THE MARY ADELAIDE NURSES.

The Workhouse Infirmary Nursing Association has H.R.H. Princess Christian for patron and Princess Mary Adelaide, Duchess of Teck, for its president, and the Hon. Mrs. J. G. Talbot and Miss Louisa Twining as vice-presidents. The general and executive committees are influential and representative ones; the hon. secretary (Miss Wilson) has devoted herself indefatigably to the welfare of the Association, with Miss Gill as her able assistant. The modest little offices at 6, Adam Street, Strand, are typical of the unpretentious method in which much good work is carried out. We strongly urge readers to call there and learn from the courteous officials of the existing need for personal and financial assistance. The Association was established in 1879 with a threefold object, namely, to raise the standard of public opinion on the whole question of workhouse nursing, to secure the appointment of trained ladies as matrons in all separate infirmaries, to train and supply nurses to workhouse infirmaries in London and the provinces.

The appreciation which the Workhouse Infirmary Nursing Association now enjoys is shown by the constant requests made for its nurses. Hence the demand for personal service from trained nurses. It is considered by the Association that a minimum period of two years' training is necessary, one year spent in a general hospital and the other in an infirmary giving suitable experience. The subscriptions to the Association are chiefly devoted to paying the expenses attendant on the training of probationers, a work which is only restricted by the limits of the funds. Now that fresh Boards of Guardians are annually added to the list of those already employing trained nurses, the limited condition of its funds has peculiar trials for an association which aims at supplying the demands of the Guardians, a work in which it has the cordial approval of the Local Government Board. Increased subscriptions are greatly wanted to meet the expenses of training an increased number of suitable women. Whilst condemnation is so liberally bestowed on infirmaries where nursing is conspicuously absent, this Association which could supply the want ought to meet with much more adequate support.

The Annual Gathering.

To this break in the year's work the Mary Adelaide nurses look forward with keen interest, but when the day of the fête arrives many are always unavoidably absent in consequence of the impossibility of leaving, even temporarily, their isolated posts of duty. This year the invitations sent out by Miss Wilson for May 22nd resulted in a large assemblage at the galleries of the Nineteenth Century Art Society. The medals and gratuities granted to various nurses for length of service were presented by her Grace the Duchess of Bedford, whose speech showed her to be sympathetically in touch with the objects of the Association. She spoke of the loneliness of the workhouse nurse's life as compared with that of the hospital worker, assuring those present of the interest felt in their work by many outside the nursing profession. The Duchess owned to a certain diffidence in speaking of work with which she was not practically acquainted, but there was no evidence of any lack of knowledge in the gracious kindliness with which she addressed her interested hearers.

Miss Twining spoke of the relief to the society which had resulted from the formation of the Association for the Northern workhouses, which now took so many unions off their hands. She read letters of regret for unavoidable absence from Lady Montague, Lady Wantage, the Hon. Mrs. Talbot, and others. Miss Twining spoke of the first annual gathering, when some five nurses met at her own house, and of the steady and encouraging growth of the Association. How much of its success is owing to Miss Twining's own personal efforts and devotion her hearers did not fail to realise.

Miss Wilson, whom Miss Twining congratulated on her restoration to health, was the recipient of a beautiful floral tribute, consisting of a basket of fine flowers and a handsome bouquet. These were presented on behalf of all the nurses by Nurse Thompson, who for fourteen, and Nurse Boddie, who for nine years, have served the Workhouse Infirmary Nursing Association. Miss Wilson acknowledged the graceful compliment thus offered her, and spoke very warmly of the good nurses with whom she had been brought into contact during the long months when she had herself been a patient. The enthusiastic welcome which her appearance on the platform provoked was sufficient evidence of the affection with which Miss Wilson is regarded.

In the course of the evening some part songs were excellently rendered by the East London Choral Union. The violin solos of Miss Edith Ardagh were much appreciated, and songs by Mr. Baddeley and Miss Gertrude Dunning gave great pleasure. Miss Gethen's narration of two of her own hospital stories also appeared to be greatly enjoyed.

Each nurse received a bunch of sweet-smelling flowers as she departed, and the party separated with many cordial expressions of mutual goodwill and grateful acknowledgements from the guests for the hospitality they had received.

Amongst the guests present were: The Duchess of Bedford, Lady Knutsford, Miss Twining, Mrs. Bonham Carter, Miss Rosalind Paget, Miss Clinton, Miss C. J. Wood, Miss de Pledge, Miss Griffiths, Miss Lamport, Dr. Humphreys, Miss Ravenhill, Miss Alexander, Miss Wesley, Miss Hannaford, Miss Styning, Dr. Savill, Mrs. S. D. Fuller, Miss Fuller Miss Haddock, and Mrs. Stanley Lane Poole.

### LONDON COUNTY ASYLUM, CANE HILL.

At the recent examination for the nursing certificate of the Medico-Psychological Association the following nurses were successful: Sophia P. Bryant, Anna L. Culot, Alice M. Dawe, Jessie Dimond, Frances Driver, Martha Finch, Grace M. Giles, Martha Harling, Anne E. Jones, Annie Meech, Bessie Partington, Elizabeth Rowe, Julia M. Sorrell, and Christina Yeats.

### Death in Our Ranks.

On April 29th, Nurse Preston died suddenly at the Ripon Hospital, Simla, of apoplexy. She was young, bright, and healthy looking. Her death was a shock to many by whom she was much esteemed in Simla.

### Notes and Queries.

### Queries.

(147) *Insane.*—I shall be greatly obliged if anyone will tell me the simplest way in which a magistrate's warrant for putting a private patient into an asylum can be obtained? It has been my duty on several occasions to accompany a case to the police-court, and the experience of waiting about in such a place with my unhappy charge is one of which I should be glad to escape a repetition.—*Trained Nurse.*

(148) *Sister.*—Please tell me whether thirteen years' experience, combined with full training in nursing, entitle me to be called sister? I have been head of large wards, and have had a staff of nurses under me.—*District Nurse.*

(149) *Probationer.*—Can you advise me of a private nurses' home where I could enter as a probationer?—*P. P.*

### Answers.

(147) *Insane (Trained Nurse).*—You would probably find a magistrate willing to see you at his own house. You should find out the right magistrate beforehand. In the country this would be specially easy. Much useful information on the subject of lunacy is given in "Helps in Sickness and to Health," published by the Scientific Press, 428, Strand.

(148) *Sister (District Nurse).*—The title is entirely one of courtesy. It is borne by the head nurses in some institutions, whilst in others it is never used. It cannot therefore be claimed merely for length of service in places where it is not the accepted title of the head or charge nurse of wards. The adoption of the word by private nurses and health lecturers has little to commend it; in the first case the term nurse is found far more acceptable to employers, whilst in the second it is more dignified for the lecturer to use her own name rather than affectedly to substitute that of, say, "Sister Belinda!"

(149) *Probationer (P. P.)*—Have you already a certificate for general nursing? In a "Home" you may learn private nursing as an addition to previous experience, but no "training" obtainable in such an institution can alone be deemed sufficient qualification for a nurse.

### Notes from Ireland.

### FETE IN AID OF SIR PATRICK DUN'S HOSPITAL, DUBLIN.

*(By Our Irish Correspondent.)*

The great fête in aid of Sir Patrick Dun's Hospital will long be remembered in Dublin for the brilliant success which has distinguished it, and for the brilliant weather to which that success has been largely due, the whole week having been remarkable for bright sunshine and pleasant breezes.

The immense concourse of people which thronged the Royal Dublin Society's buildings and grounds every afternoon and evening bore witness to the public's appreciation. The record of attendances, beginning with over 8,000 on Tuesday, and swelling to over 19,000 on Thursday, speaks for itself, and must be grateful indeed to the promoters of the fête, and to all interested in the welfare of the hospital, to which the proceeds will be devoted.

His Excellency Lord Houghton, who was received at the main entrance by Dr. Walter Smith, President of the Royal College of Physicians, Ireland, with several other distinguished members of the medical profession, and of the "Jervis" Committee, formally opened the fête on Tuesday afternoon, making a short speech, in which he expressed the pleasure it gave him to do so, and his hope that the success of "Jervis" would at least equal that of "Kosmos" and of "Araby." His Excellency, who was accompanied by his daughters and a distinguished party, then visited several of the stalls, spending a considerable time in his progress through the bazaar, and, no doubt, a corresponding amount of money.

The main hall of the building was cleverly metamorphosed for the occasion into a glorified edition of an old town, the surrounding stalls representing different specimens of ancient architecture, principally Irish, while the centre was devoted to the flower market and the most bewitching of tea gardens. This is a new improvement, as at all foregoing fêtes this space has been occupied by a wooden enclosure for children's dances, &c., wisely transferred by the "Jervis" Committee to the South Hall with an appreciation of the value of first impressions. Certainly the alteration was justified by the result, for nothing could have been more effective than the design of the stalls on one side of the hall viewed from the other, especially at night, when the crumbling walls and ivy-wreathed towers were absolutely realistic. The goods on sale at the stalls were of the most enticing description, and the business done in this, as in all other departments, was exceedingly good. Very large sums were also realised by raffles, but in these enormous fêtes the attractions of the "bazaar proper" invariably take a secondary place, and it is upon the various amusements that the promoters depend for the realisation of golden dreams. These amusements at "Jervis" comprised a water-chute—a novelty on this side of the Channel, and immensely popular—balloon ascents, fancy and international dances, theatricals, concerts, and variety entertainments (professional and amateur), a café chantant, a loan museum, a smoking divan, switchbacks, &c., of every variety. Athletic sports and fireworks were held in the jumping enclosure, whilst facilities for the enjoyment of afternoon tea were combined with the amusement of watching the ever-changing and always entertaining throng. Last, but not least, an excellently managed dining hall, where those who required more substantial refreshments were treated in every respect as well as at a first-rate restaurant. Add to all this the zest and spirit with which everyone, young and old, gentle and simple, entered into the humours of the fête—for we Irish cannot be accused of "taking our pleasure sadly"—and Hospital readers will have as good an idea of "Jervis week" as can be conveyed to anyone not fortunate enough to participate in its enjoyments.

### Where to Go.

Holy Trinity Club Rooms, Shoreditch.—Children's Geranium Club and Flower Show will be opened by Her Royal Highness the Duchess of Albany at quarter-past three on June 13th.

Anyone who likes clean and wholesome humour cannot fail to enjoy the new play at the Comedy Theatre. "The Prude's Progress" is fresh and amusing from first to last, and it well deserves the cordial reception accorded to it. Jerome K. Jerome and Eden Phillpotts are to be congratulated on the success of their joint plot.

Cassell's Black and White Exhibition.—This year, more than ever, we are brought face to face with the fact that illustration is not only showing a very marked improvement, but is fast asserting itself as a leading power in art. This exhibition at Cutlers' Hall brings the fact dominantly before our eyes as it has not been brought before. Many among the 440 works here exhibited are of extreme beauty, and worth more than the passing notice space compels us to give. Black and white drawings have an expression of their own, and convey to our minds, in some instances here presented, as full a suggestiveness as any colour drawing could do. In Mr. Muckley's, for example, and in Mr. Haite's it is hard to convince oneself that the colour of the landscape which we seem to see is, after all, our own imagining. In Mr. C. Wilkinson's "In the Cornfields" (No. 321) this is also the case; the ripe sheaves of corn look as golden as any seen on an autumn day, and yet the medium employed is only that of light and shade. And the same must be said of composition pictures, of which there are a greater number. "Good Night" (No. 159), by Miss M. Dicksee, especially, perhaps, proves this to be the case, where she shows us two small children in bed in the absolute sleep which childhood alone completely represents. Mr. Wyllie gives us a charming study of sea life in "The Lifeboat" (No. 163), a study full of life and suggestiveness. A clever sketch of the Parnell Commission by Mr. F. W. Wilson, R.I., is here, too, and Mr. Railton's pictures of the Royal College of Surgeons and the Record Office are good specimens of pen and ink work. The exhibition is a decidedly interesting one, and well repays one a visit to the City to enjoy it. Its doors are open to us only from May 22nd to June 7th.

Exhibition of the Nineteenth Century Art Society.—The summer exhibition of this society is now opened at the Conduit Street Galleries The principal object of the society is, we are told, to give greater facilities for the showing and sale of works by recognised and rising young artists, an object wherein at once lies the strength and the weakness of the exhibition. Although it is an excellent opportunity for those artists who are described as recognised and rising, yet, somehow, the walls are stamped with a certain amateurness. All through one notices this: it is an exhibition of the rising more than of the finished artist. But there are some pictures which are indeed redeeming features, and it is of these we would speak as being striking through their excellence. Take for instance the big central picture in the large gallery, No. 93. A place of honour is awarded it, and justly enough. Mr. Charles Pettafor calls this canvas "The Time of the Singing of Birds is Come;" it is in reality a study of birch trees, and an extremely lovely study, too. They are painted in with a light suggestive touch, but not too transparent a one. Portraiture is not the strong point of the exhibition; there are several specimens, but none of them good ones Composition is best exemplified in Mr. Arthur Vernon's "Betrayed Secrets," certainly the best subject picture although not the largest. The large central canvas on the opposite wall (No. 150, "Found") by Mr. Heydeman might be catalogued Scene-painting; it is theatrical, but not artistic. Turning to the water-colours in the next room we find ourselves on the whole in a less amateurish company; some are remarkably good. Miss Agnes Kemp gives us three examples of her brush—two landscapes and a portrait. Miss Kemp should paint on a larger scale, the size of her pictures being their only fault, for they are altogether out of the ordinary run of woman's work. No. 229, "A Study of a Head," is striking in its tenderness and breadth of treatment, and the pathos in the expression of the old woman's face is haunting; throughout the picture there is neither a line too much nor too few. This is the thirty-fourth exhibition of the society, and though the gallery is a small one there is some really good work present.

### Everybody's Opinion.

[Correspondence on all subjects is invited, but we cannot in any way be responsible for the opinions expressed by our correspondents. No communications can be entertained if the name and address of the correspondent is not given, or unless one side of the paper only be written on.]

### A CORRECTION.

A correspondent draws attention to the fact that the present Midwives Bill was "almost entirely drawn up by medical men, of which it shows plenty of evidence," but not by the "Midwives' Board," as inadvertently stated last week.

### *RE* PRACTICAL POINTS.—HOSPITAL FLOORS.

Miss Agnes Bradwell, matron of the Hospital at Louth, Lincolnshire, writes: May I suggest to your correspondent that if no money can be spent on her corridor floor, to have used for scrubbing J. M. Smith and Co.'s disinfecting cleansing powder, Hampton Chemical Works, Borough Road, S.E. May be obtained through any chemist. It is admirable for cleaning board floors, and economical. No soap or soda is required, labour is lightened, the wood is hardened and altogether improved in appearance. Our ward floors were in a very unsatisfactory state a year ago, with twenty years' wear and scrubbing. We have used nothing else for the last twelve months, and they are not like the same floors, so much firmer and clearer, with less scrubbing.

### A WARNING TO NURSES.

"Nurse M. W." writes: I think that "A Nurse" was quite justified in not following the man she mentions. Once inside a house, to withdraw, as "Nurse W." suggests, might not be an easy matter. Surely if the man's wife were ill he could get assistance without calling in a nurse from the street. There are plenty of hospitals and private and district nurses to meet all demands, and the nearest doctor would have given information and help. I am not particularly mistrustful but I certainly believe in due caution, especially in London. "Nurse W." says that nurses should have no thought of "self." I consider that remark rather uncalled for. What dangers are nurses not subject to? If they thought of themselves they could not face them. Not only dangers, but scenes of suffering have to be gone through which are often so trying. I think we owe some thanks to the nurse for her warning.

### TRAINING AND TITLES.

"District Nursing Sister" writes: I wonder what the nursing world in general thinks of the enclosed cutting? After so much has been done to secure a thorough training for district nurses it seems impossible to believe that any one can countenance this "six months' course."

[Our correspondent's enclosure is a Press report of the Essex Cottage Nursing Association, and the paragraph to which "District Nursing Sister" refers runs as follows: "Ten women have either received or are receiving a six months' course of training, of these Nurse Moule is on the staff for sending into affiliated parishes not having a resident nurse; . . . whilst the six others will be opened for engagements in June, July, September, October, November, and December respectively." As a fully-trained district nurse we can understand that our correspondent is dismayed at reading of a woman being sent with the misleading title of Cottage Nurse into a parish not having a resident nurse, after she has gone through only a six months' course."—Ed. *T.H.*]

### ROYAL BRITISH NURSES' ASSOCIATION.

The quarterly meeting of the General Council will be held at five p.m. on Friday, July 12th, at 17, Old Cavendish Street.

### Small-pox in the Desert—A Nurse's Experience.

(Communicated.)

A very sad occurrence happened on board the ss. *Coromandel* on her homeward voyage last March. Soon after leaving Calcutta, Small-pox broke out on board and two of the patients were landed at Colombo. The disease was very prevalent in Calcutta last winter, and it was said that 6,000 deaths occurred in a month. After Colombo a lady well known in Calcutta fell sick with confluent or malignant small-pox, and no one would go near her, though she had many friends on board. Fortunately for Mrs. Milne, Miss Wildman, late lady superintendent of the Ramsay Hospital, was on board. She herself being down in her cabin, though luckily not with small-pox, was unaware of what was going on until the medical officer mentioned it. She went at once to the patient and stayed with her two days and two nights on board till the ship put in to Aden, where both ladies were landed, the P. and O. refusing to take them on. At first no one would receive them, but finally they were taken to an isolated place some three miles from Aden and a single-roomed hut was given to them. Mrs. Milne died three days later. The only assistance Miss Wildman had in her terrible vigil at Aden was from a native compounder. The sweeper (coolie) sent out to her was overcome with abject terror, and quickly deserted. The civil surgeon visited them daily. The poor lady went blind before she died, which was, perhaps, merciful. Her fever at the end was great, her temperature first 106 deg., then 108·2 deg., and just before she died it rose to 109·4 deg. The next morning she was buried, Miss Wildman having passed the night alone in the solitude of the Aden desert, seated on a stone outside the hut till day-break. Before she could get away from a spot fraught with such sad memories everything had to be burnt—clothes and all else Miss Wildman had with her. Eventually she was taken to the European General Hospital, where she was given two rooms to herself, and there at last obtained much-needed rest. After due quarantine, she received much kindness from the European residents in Aden, and left on April 7th by the ss. *Oriental* for London.

### Presentations.

East Lancashire Infirmary, Blackburn.—The nursing staff of this hospital have presented Dr. C. E. Maude (on his resignation of the post of house surgeon) with a very handsome travelling bag, fully fitted up, in recognition of the interest he has taken in the improvement of the nursing work in the infirmary, and also of the personal care he has bestowed on the training of the nurses. Dr. Maude has acted as lecturer to the nurses for the past two years, and his resignation is much regretted

Miss Amy Hughes, on resigning the post of Superintendent of the Central Training Home for the Queen's Jubilee Institute, was presented with a handsome carriage clock and a silver tea-pot by sixty-eight of the nurses who have received their district training under her. The gift was accompanied by cordial good wishes for Miss Hughes' success in her new work at Bolton, as well as by many regrets at her departure from the post which she held with such remarkable ability at Bloomsbury.

### COTON HILL HOSPITAL, STAFFORD.

On May 6th and 9th, Dr. Whitcombe, medical superintendent of the Birmingham Borough Asylum, conducted the examinations at which the following attendants obtained the certificate of proficiency in nursing of the Medico-Psychological Association:—Nelly Dodd, Lizzie Jane Jones, Annie Newboult, Annie Pitt, Sarah Ann Silvester, Henry Arthur Beardsall, Henry Horton, Thomas Robinson, and Alfred Henry Robinson.

